# Hybrid surgical technique for open abdominal aortic aneurysm repair in the setting of severe iliac artery calcification

**DOI:** 10.1016/j.jvscit.2023.101141

**Published:** 2023-03-05

**Authors:** Halli Krzyzaniak, Griffins Misati, Mark Rockley, Kenton Rommens

**Affiliations:** a Cumming School of Medicine, University of Calgary, Calgary, AB, Canada; b Division of Vascular Surgery, Libin Cardiovascular Institute, University of Calgary, Calgary, AB, Canada; c Department of Vascular Surgery, University of Ottawa, Ottawa, ON, Canada; d Calgary Aortic Program, Libin Cardiovascular Institute, University of Calgary, Calgary, AB, Canada

**Keywords:** Aortic aneurysm, Iliac calcification, Open repair

## Abstract

In the present report, we describe the case of a patient with an infrarenal abdominal aortic aneurysm that had been incidentally noted on an imaging study. Treatment decisions for this case were complicated by the presence of a hostile infrarenal aortic neck and significant bilateral iliac artery circumferential calcification, precluding iliac artery clamping and standard distal anastomotic techniques. We performed a hybrid surgical procedure, deploying bilateral iliac stent grafts into the distal aneurysmal aorta and sewing our aortic graft to the proximal extent of these stents. The findings from the present case add to the previously reported techniques of hybrid surgical management of abdominal aortic aneurysms with iliac disease and expand the technique to a larger application.

## Case report

A 77-year-old man was admitted with an incidentally found infrarenal abdominal aortic aneurysm (AAA). His medical history included coronary artery disease, hypertension, type 2 diabetes mellitus, and a 45 pack-year smoking history. He had no symptoms related to the aneurysm or occlusive disease. The patient provided written informed consent for the report of his case details and imaging studies.

**Fig 1:**
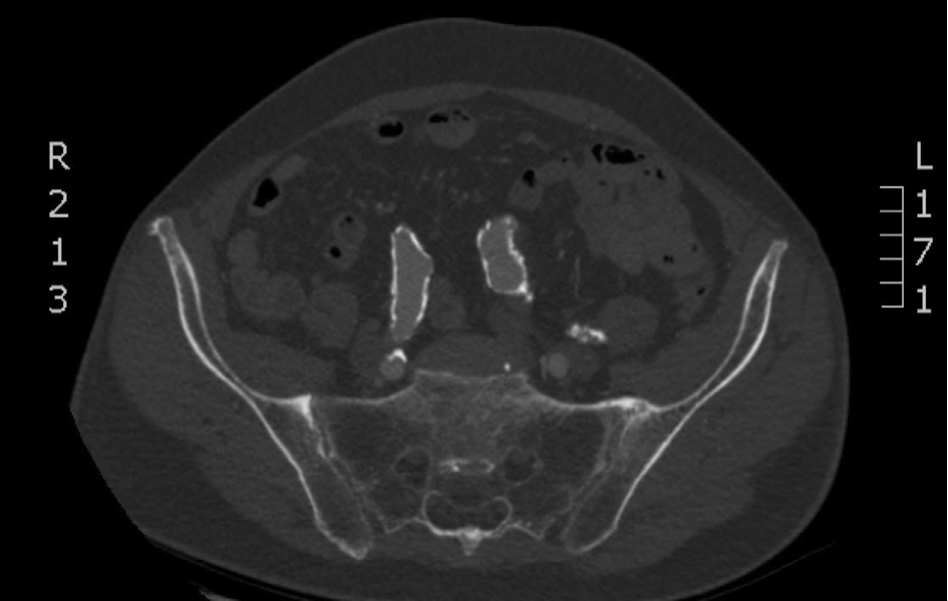
Axial computed tomography angiogram showing significant bilateral iliac artery calcification.

A computed tomography angiogram revealed a fusiform infrarenal AAA measuring 7.8 cm in maximal diameter. The neck morphology was conical with a 4.4-mm infrarenal neck measuring 23 mm in diameter, which subsequently dilated to 33 mm. Circumferential calcification was present from the aortic bifurcation to the external iliac arteries bilaterally (Fig 1). His left common iliac artery was mildly ectatic and had splayed his aortic bifurcation to a diameter of 48 mm.

Given the morphology of his infrarenal neck, he did not qualify for standard endovascular aneurysm repair (EVAR) according to the indications for use (IFU). Fenestrated EVAR was not considered because of the size of the AAA and the 4- to 6-week estimated wait time to receive a custom graft. A tube graft was not possible owing to the calcification and diameter of his aortic bifurcation. Preoperatively, our patient was cleared for open repair by our internal medicine colleagues, and, after a discussion of the risks and benefits, he elected to proceed.

We conducted the operation in a hybrid vascular operating room with the patient on an endovascular table. A midline laparotomy for exposure of the infrarenal aorta was performed. After achieving circumferential control of the proximal aortic neck, dissection continued to the common iliac arteries. These were found to be extensively calcified and not amenable to clamp control or as tissue for an anastomosis. Thus, we decided to proceed with a hybrid approach using bilateral endovascular iliac limbs in the common iliac arteries with compliant balloons for distal control.

Percutaneous femoral access was obtained using ultrasound. After systemic heparinization, serial dilations were performed up to 12F DrySeal sheaths (W.L. Gore & Associates, Flagstaff, AZ). A retrograde angiogram identified the origin of the internal iliac arteries. A 16 × 16 × 9.5-mm Excluder limb (W.L. Gore & Associates) was deployed slightly proximal to the right iliac bifurcation and extending above the aortic bifurcation into the aneurysm sac. Subsequently, a 16 × 18 × 9.5-mm limb was placed in the left common iliac artery in the same fashion. Balloon molding was performed with Coda balloons (Cook Medical Inc, Bloomington, IN), which were left in the distal common iliac stents.

**Fig 2:**
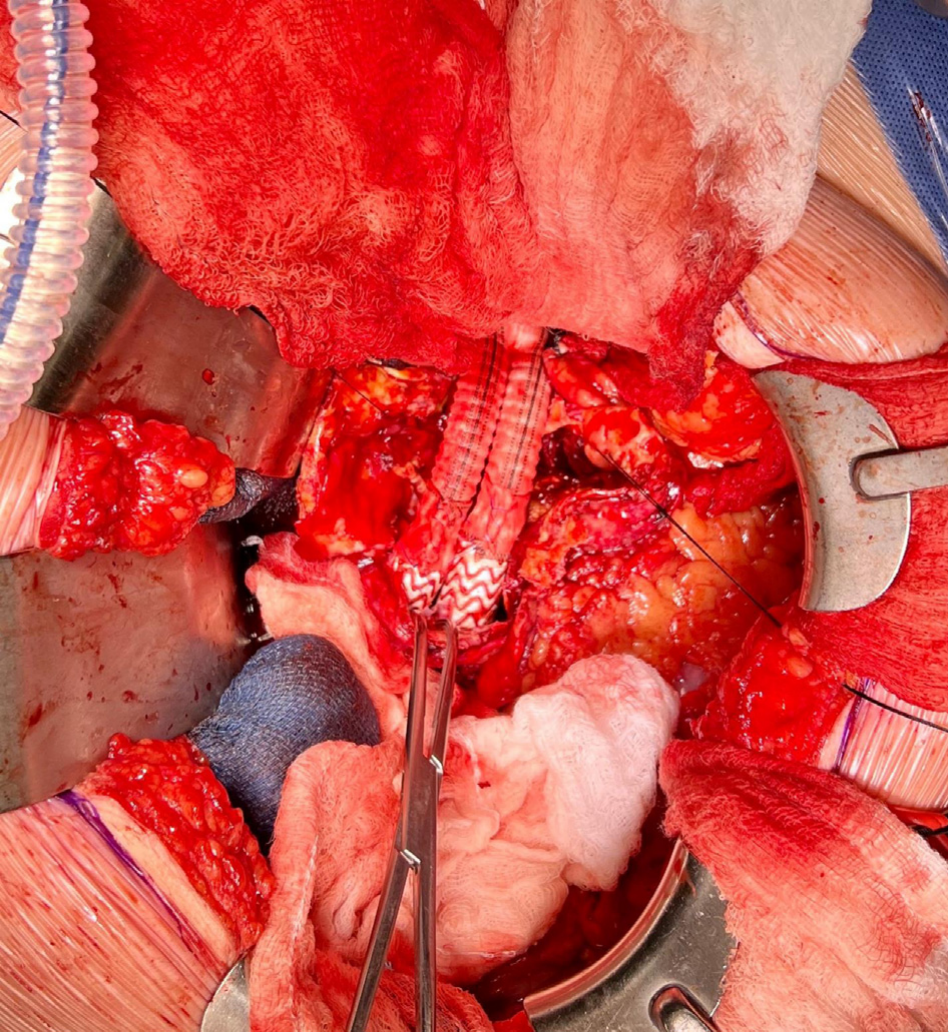
Intraoperative photograph showcasing the distal graft to stent anastomosis with Dacron cerclage.

An infrarenal aortic clamp was placed, followed by inflation of the balloons in the iliac stents. The aneurysm sac was opened, thrombus was removed, and the lumbar arteries were oversewn. A 20 × 10-mm bifurcated Dacron graft (Terumo Medical Corp, Somerset, NJ) was selected, and the proximal anastomosis was completed using 3-0 Prolene sutures. The serrated proximal edges of the iliac stents were removed, and the Dacron graft was trimmed. An end-to-end anastomosis was performed between the Dacron graft limbs and the iliac stents with 4-0 Prolene sutures. To ensure adequate graft-to-stent hemostasis, the remaining Dacron fabric was used as a cerclage with double-layer 4-0 Prolene sutures (Fig 2).

All clamps and balloons were removed, and we found excellent outflow through the femoral artery sheaths. Predeployed Perclose ProGlide suture-mediated closure systems (Abbott Cardiovascular, Plymouth, MN) were used to achieve hemostasis after removal. Protamine was administered, the aneurysm sac was sutured over the graft using 2-0 Vicryl, and the abdomen was closed. The estimated blood loss for the operation was 1500 mL, with 690 mL autotransfused back to the patient through cell salvage.

Postoperatively, his pain was controlled with an epidural catheter before transitioning to intravenous and then oral analgesics. He experienced postoperative bowel ileus requiring a nasogastric tube, which was removed once he had begun passing flatus, and he progressed to a normal diet with no further issues. He was discharged on postoperative day 8 in good condition.

A computed tomography angiogram was completed before discharge. The bifurcated graft and iliac stent grafts were patent, with no evidence of an endoleak or kinking. He was seen for follow-up at 1 month postoperatively and had returned to his baseline level of function, with no operative concerns.

## Discussion

The findings from the present case demonstrated good operative success with a hybrid approach to treating AAAs in the context of a hostile infrarenal neck and significant iliac artery calcification. Intraoperative interrogation of our patient's iliac arteries did not identify a suitable distal clamp site; therefore, the decision was made to proceed with retrograde bilateral iliac stents. The bifurcated Dacron graft was sewn in standard fashion at the proximal anastomosis, with the distal limbs sewn to the proximal iliac stent grafts.

Endovascular repair of AAAs has increased owing to its less invasive nature and lower perioperative mortality.^1^, ^2^, ^3^, ^4^ However, EVAR has been associated with higher reintervention rates and aneurysm-related mortality, with the initial benefit of decreased mortality lost by 6 months to 1 year postoperatively.^2^^,^^5^, ^6^, ^7^ Additionally, endovascular graft use is limited by anatomic considerations, because nonadherence to the IFU has been associated with adverse events.^8^^,^^9^

Our patient's anatomy did not meet the IFU owing to the reverse conical configuration of his infrarenal neck. It has been shown that this is the strongest risk factor for a type Ia endoleak, which will require additional interventions.^10^^,^^11^ Thus, we elected to proceed with open repair after a long discussion with the patient regarding the potential intraoperative difficulties.

Although good results using endovascular strategies to treat both aortoiliac occlusive disease and aneurysmal disease have been reported in a case series,^12^ few studies have reported on hybrid procedures for patients requiring open repair. Touma et al^13^ described two patients who had undergone open AAA repair via a retroperitoneal approach with a right iliac graft used at a distal anastomotic site. The first patient had presented with a ruptured juxtarenal AAA, and a right iliac limb was deployed in antegrade fashion after the surgeons had encountered nonsuturable common iliac arteries. The second patient had undergone open retroperitoneal repair of a large suprarenal aneurysm. Before aortic exposure, a right iliac stent was deployed in the common iliac artery in retrograde fashion, with the proximal end floating within the distal aneurysm.^13^ In both patients, the left limb was sutured to the native circulation.

Another group described their results using a staged hybrid procedure to treat juxtarenal AAAs with an associated iliac artery aneurysm.^14^ The five patients included had undergone initial endovascular management of their iliac aneurysm. Open repair of the juxtarenal AAA was performed the next day, with distal anastomoses sewn between the aortic graft and previously placed iliac stents. Two patients had experienced transient postoperative gluteal claudication; however, the investigators reported the technique was technically successful.^14^

The findings from the present case add to the previously described hybrid techniques for managing concomitant iliac disease and AAAs. Our patient was treated via a midline laparotomy with bilateral retrograde iliac stent grafts for distal anastomosis and hemostatic control. This differs from previous studies, which had used a retroperitoneal approach^13^ or a two-stage procedure,^14^ and extends the potential applications for a hybrid approach to AAA management.

## Conclusions

We have demonstrated good technical and clinical success using a hybrid surgical technique to facilitate open AAA repair in the setting of heavily calcified iliac arteries using bilateral iliac stent limbs and graft-to-stent distal anastomoses.
